# Features of asthma which provide meaningful insights for understanding the disease heterogeneity

**DOI:** 10.1111/cea.13014

**Published:** 2017-09-15

**Authors:** M. Deliu, T. S. Yavuz, M. Sperrin, D. Belgrave, U. M. Sahiner, C. Sackesen, O. Kalayci, A. Custovic

**Affiliations:** ^1^ Division of Informatics, Imaging and Data Sciences Faculty of Biology, Medicine and Health University of Manchester Manchester UK; ^2^ Department of Pediatric Allergy Gulhane School of Medicine Ankara Turkey; ^3^ Department of Paediatric Allergy Children's Hospital University of Bonn Bonn Germany; ^4^ School of Medicine Pediatric Allergy Unit Koc University Istanbul Turkey; ^5^ Pediatric Allergy and Asthma Unit Hacettepe University School of Medicine Ankara Turkey; ^6^ Department of Medicine Section of Paediatrics Imperial College London London UK

**Keywords:** allergic sensitization, asthma, childhood, cluster analysis, endotypes, phenotypes, severe asthma

## Abstract

**Background:**

Data‐driven methods such as hierarchical clustering (HC) and principal component analysis (PCA) have been used to identify asthma subtypes, with inconsistent results.

**Objective:**

To develop a framework for the discovery of stable and clinically meaningful asthma subtypes.

**Methods:**

We performed HC in a rich data set from 613 asthmatic children, using 45 clinical variables (Model 1), and after PCA dimensionality reduction (Model 2). Clinical experts then identified a set of asthma features/domains which informed clusters in the two analyses. In Model 3, we reclustered the data using these features to ascertain whether this improved the discovery process.

**Results:**

Cluster stability was poor in Models 1 and 2. Clinical experts highlighted four asthma features/domains which differentiated the clusters in two models: age of onset, allergic sensitization, severity, and recent exacerbations. In Model 3 (HC using these four features), cluster stability improved substantially. The cluster assignment changed, providing more clinically interpretable results. In a 5‐cluster model, we labelled the clusters as: “Difficult asthma” (n = 132); “Early‐onset mild atopic” (n = 210); “Early‐onset mild non‐atopic: (n = 153); “Late‐onset” (n = 105); and “Exacerbation‐prone asthma” (n = 13). Multinomial regression demonstrated that lung function was significantly diminished among children with “Difficult asthma”; blood eosinophilia was a significant feature of “Difficult,” “Early‐onset mild atopic,” and “Late‐onset asthma.” Children with moderate‐to‐severe asthma were present in each cluster.

**Conclusions and clinical relevance:**

An integrative approach of blending the data with clinical expert domain knowledge identified four features, which may be informative for ascertaining asthma endotypes. These findings suggest that variables which are key determinants of asthma presence, severity, or control may not be the most informative for determining asthma subtypes. Our results indicate that exacerbation‐prone asthma may be a separate asthma endotype and that severe asthma is not a single entity, but an extreme end of the spectrum of several different asthma endotypes.

## 1 INTRODUCTION

The evidence is mounting that asthma is an umbrella diagnosis for a collection of distinct diseases (endotypes), with varying phenotypic expression of characteristic symptoms (ranging from wheezing and shortness of breath, to cough and chest tightness), and accompanying variable airflow obstruction.^1^, ^2^, ^3^ It is important to make a clear distinction between asthma phenotypes (which are observable and measured characteristics of the disease)^4^ and asthma endotypes (which is a term that refers to the subtype of the disease with a clearly defined underlying mechanism).^1^, ^2^, ^5^ It is of note that similar symptoms and observable features can arise through different pathophysiological mechanisms and that consequently different endotypes may have similar, or even the same phenotype. Identifying true endotypes of asthma and their underlying mechanisms is a prerequisite for achieving better mechanism‐based treatment targeting, and ultimately delivery of genuinely stratified medicine in asthma.^5^ However, although the current consensus in the medical community is that different asthma endotypes do exist, there is little agreement on what these are and how best to define them.^6^

Approaches utilized in the search for asthma endotypes have ranged from investigator‐led pattern identification, in the clinical setting, to supervised and unsupervised statistical modelling techniques that utilize large amounts of data and computer algorithms to find the latent (hidden, unknown a‐priori) patterns of observable features (such as symptoms, medication use, allergic sensitization, lung function), either in cross‐sectional studies^7^, ^8^, ^9^, ^10^ or over time. Data‐driven approaches allow interrogation of data without imposing a‐priori hypotheses, hence eliminating investigator bias and enabling novel hypotheses to be generated.^6^ In most previous studies which used such approaches, the selection of variables used for subtype discovery was either pre‐determined by clinical advice,^7^, ^9^, ^11^ or by the use of statistical data reduction techniques such as principal component analysis (PCA).^8^, ^12^, ^13^ Although valuable information has been gained, and there was some (but not complete) resemblance between the results, most studies reported different disease clusters; several recent reviews have summarized these findings.^14^, ^15^, ^16^, ^17^, ^18^ These inconsistencies may be explained by the inherent heterogeneity among different populations, the differences in clustering techniques used, the lack of consistency in selecting variables, their encodings and transformations, or the use of excessive numbers of variables which may result in subtype “signals” being drowned in the noise.^19^

When selecting the variables for unsupervised analyses, the investigators rely on the data which are available (eg in birth cohorts^10^, ^20^, ^21^ or studies of adults and children with established disease).^7^, ^8^, ^9^ In most clinical studies, the assessment and monitoring of study participants focuses on measures which aim to ascertain asthma presence, severity, control, and responsiveness to treatment. We hypothesize that these may not necessarily be the variables or features which are most informative for the discovery of disease endotypes. We propose that a careful synergy of data‐driven methods and clinical interpretation may help us to better understand the heterogeneity of asthma and enable the discovery of true asthma endotypes. In this study, we aimed to ascertain whether a framework for data interrogation which utilizes an integrative approach that brings together the data and biostatistical expertise, with a clinical expert domain knowledge and clinical experience, can facilitate the identification of stable and clinically meaningful asthma subtypes.

## 2 METHODS

### 2.1 Study design, setting, and participants

We used anonymized data from a cross‐sectional study which recruited children with asthma aged 6‐18 years from two hospitals in Ankara, Turkey (Hacettepe and GATA University Hospitals); the study is described in detail elsewhere.^19^, ^22^, ^23^ Briefly, children who presented to the Paediatric Allergy and Asthma Units completed skin prick tests, spirometry, and measurement of bronchodilator reversibility (BDR). Among children with a negative BDR test (<12% increase in FEV_1_ following administration of 200 μg of albuterol), airway hyperresponsiveness (AHR) was assessed using methacholine or exercise challenge test.

Asthma was defined as all three of the following: (i) physician‐diagnosed asthma; (ii) current use of asthma medication; and (iii) either BDR or AHR (positive methacholine or exercise challenge test). Children with other known systemic disorders such as cystic fibrosis or immunodeficiency, and those who had a severe exacerbation requiring systemic corticosteroids or hospital admission within the previous 4 weeks were not included.

### 2.2 Data sources/measurements

We recorded a total of 47 variables for each study participant; of those, 45 were used in the analysis (Table S1).

#### 2.2.1 Symptoms, exacerbations, and prescribed medications

A modified ISAAC questionnaire was interviewer‐administered to ascertain the age of onset, the presence of asthma‐related symptoms within the past 4 weeks, the number of asthma exacerbations within the past year, and hospitalizations for acute asthma (ever).

#### 2.2.2 Asthma severity

Categorized as mild, moderate, or severe based on GINA guidelines ( www.ginasthma.org); a detailed description is published elsewhere.^22^ Briefly, we allocated patients to severity group based on the assessment of clinical symptoms before the treatment was initiated; when the patient was already receiving treatment, the severity was assigned based on the clinical features and the step of the daily medication regimen (for details, please see online supplement).

#### 2.2.3 Lung function

We performed spirometry, methacholine, and/or exercise challenge tests according to ATS/ERS guidelines;^24^, ^25^, ^26^ FEV_1_ (% predicted), FVC, FEV_1_/FVC, and FEF_25‐75_ were recorded.^27^

#### 2.2.4 Allergic sensitization

We carried out skin prick testing to a battery of allergens including dust mite, tree, grass and weed pollens, moulds, cat, dog, cockroach, and horse. Weal 3 mm greater than negative control was considered a positive reaction. We also measured total serum IgE.

#### 2.2.5 Objective measurements

Height, weight, body mass index (BMI; standardized for age and growth and sex), and blood eosinophils.

### 2.3 Statistical methods

All analyses were performed in R software ( www.r-project.org/).^28^ For a detailed description of statistical methods, please see the online supplement. Briefly, we performed a hierarchical cluster analysis (HC) using three different models:

1. *HC after PCA dimensionality reduction:* We first performed PCA on all variables in the data set, and then carried out HC using principal components with eigenvalues >1.
2. *HC using all available variables:* We performed HC on raw data, without removing or modifying any of the variables.
3. *Identification of a subset of potentially important features, and clustering using the informative subset:* The results of the first two models were reviewed by clinical experts to identify features (domains) in the data set which may drive cluster allocation. We then used these informative features in a further HC.

Cluster stability was tested with bootstrapping methods. The data were resampled, and the Jaccard similarities of the original clusters to the most similar clusters in the resampled data were computed. The mean of the similarities was used as an index of stability, and a mean greater than 0.75 was deemed as stable.^29^

We used logistic regression to identify variables which differed between the clusters.

All study procedures were carried out in accordance with a protocol previously approved by the Ethics Committee of Hacettepe University Ethics committee (# FON 02/24‐1) and the Ethics Committee of Gulhane School of Medicine (05.06.2013/21). All parents provided written informed consent, and children provided assent for the study procedures.

## 3 RESULTS

### 3.1 Participants and descriptive data

The study population comprised of 613 asthmatic children (64% male, median age 9 years, 49% with physician‐diagnosed allergic rhinitis, 39% exposed to tobacco smoke, 59% atopic, all receiving SABA as needed, 61% receiving ICS, 15% experiencing 2 or more asthma exacerbations in the previous year, with mean FEV_1%_ predicted of 87%). The characteristics of the study population are shown in Table 1. Asthma was classified as mild, moderate, or severe in 78%, 20%, and 2% of cases, respectively.

**Table 1:** Demographic characteristics of the study population

N = 613	Mean (SD) % (N)
Age at follow‐up (y)	9 (3.0)
Sex (male)	64% (392)
BMI	18.4 (3.6)
Age of asthma onset (years)	5 (3.4)
Family history of asthma (yes)	30% (184)
Exposure to tobacco smoke (yes)	39% (240)
Skin prick test positivity	59% (361)
FEV_1%_ predicted	87 (14.3)
FVC % predicted	96 (15.1)
FEV_1_/FVC (%)	86 (7.0)
Bronchodilator reversibility (%)	17.1 (12.9)
Total IgE (kU/L)	228 (458)
Blood eosinophil (%)	4.4 (3.5)
Asthma severity	
Mild	78% (476)
Moderate	20% (126)
Severe	2% (11)
Using regular ICS	61% (375)
ICS dose >400 mcg	18% (113)
Using regular Montelukast	8% (51)
Using regular controller medication (ICS/LABA and/or Montelukast)	63% (385)
Using regular ICS/LABA	8% (51)
2 or more asthma attacks within the last year	15% (95)
2 or more hospitalizations for asthma ever	5% (29)
Presence of rhinitis	49% (302)
Presence of eczema	6% (37)

BMI, body mass index; FEV1, forced expiratory volume in 1 s; FVC, forced vital capacity; ICS, inhaled corticosteroid dose represented as BDP equivalent; LABA, long‐acting beta_2_‐agonist; SABA, short‐acting beta_2_‐agonist. Continuous variables are given as mean and standard deviation, and binary variables are given as percentages with absolute values.

### 3.2 Data‐driven analyses: Dimensionality reduction vs clustering using all available variables

#### 3.2.1 HC after dimensionality reduction

Dimensionality reduction using PCA identified 19 components with eigenvalues above 1, which accounted for 73% of the variance within the data set. The correlation matrix of the variables is shown in Fig. S1. Variables describing atopy correlated highly, as did those relating to lung function and medication use. Table S2 shows the eigenvalues and variance explained by 19 components, and Table S3 the variable contribution/loading to each of the first five components.

A five‐cluster model in HC after PCA dimensionality reduction provided the most clinically interpretable results. Table S4 shows clinical features/variables which differed across the clusters. Based on their dominant features, we labelled the clusters as: Cluster 1 (n = 102), “Moderately‐severe asthma with poor lung function, high symptom burden and medication use”; Cluster 2 (n = 70), “Middle school‐age onset, predominantly male, with high symptom burden despite normal lung function”; Cluster 3 (n = 117), “Late‐onset, multiple sensitization, mild asthma with diminished lung function”; Cluster 4 (n = 149), “Early‐onset atopic mild asthma, predominantly female”; and Cluster 5 (n = 175), “Mild atopic asthma.” Children in Cluster 1 had the lowest lung function, with FEV_1_ 21% lower compared to those in Cluster 5. Clusters 2 and 3 comprised of predominantly boys, while those in Cluster 4 were mostly girls. Allergic comorbidities were significant features of Cluster 3.

#### 3.2.2 HC using all available variables

As in the previous model, a five‐cluster solution provided the most clinically interpretable results. However, the clusters were different, both in terms of clinical characteristics and the number of children in each cluster. Table S5 highlights clinical features and variables which differed across the clusters. We labelled the clusters as: Cluster 1 (n = 168), “Early‐onset severe asthma, predominantly female”; Cluster 2 (n = 100), “Late‐onset mild atopic asthma”; Cluster 3 (n = 103), “Moderate‐severe atopic asthma”; Cluster 4 (n = 223), “Mild non‐atopic asthma, predominantly male”; and Cluster 5 (n = 19), “Middle‐school age of onset, atopic, with frequent exacerbations.” Children in Cluster 3 had the poorest lung function (mean FEV_1_ 72.6%), Cluster 2 was associated with allergic comorbidities, and Cluster 5 was predominantly associated with exacerbations (Table S5).

#### 3.2.3 Cluster stability

Cluster stability was generally poor for both models, with HC on principal components producing only one stable cluster (Cluster 1), and HC using all available data producing two stable clusters (Clusters 2 and 5).

### 3.3 Blending the data and biostatistical expertise with clinical expert domain knowledge

#### 3.3.1 Identification of stable features which distinguish the clusters

We first compared the subject allocation between the two analyses to ascertain the overlap which could indicate similarity (Table S6). However, there was little overlap (apart from one cluster pair, Cluster 5 in HC after PCA, and Cluster 4 in HC using all variables). We therefore proceeded with the comparison of the characteristics of clusters which we identified using the two methods. Clinical domain experts reviewed the results (Tables S4‐S6) to highlight features and variables which characterized each cluster, and similarities and differences between the clusters (Table S7). We then used clinical expert domain knowledge and experience to identify four disease features/domains common to each cluster in both models: (i) age of onset; (ii) allergic sensitization; (iii) asthma severity; and (iv) recent exacerbations. We assigned these four features as an “informative set,” and proceeded to ascertain whether using this set may help distinguish asthma subtypes.

#### 3.3.2 HC using the informative set of features

In HC using this informative subset of features, a five‐cluster solution provided the most clinically interpretable results. Compared to previous analyses, the cluster assignment changed, but the cluster stability improved substantially (Table S8, bootstrap mean ≥ 0.99). Table 2 shows clinical features which differed across the clusters. Based on the dominant features of each cluster, we labelled them as: Cluster 1 (n = 132), “Difficult asthma”; Cluster 2 (n = 210), “Early‐onset mild atopic asthma”; Cluster 3 (n = 153), “Early‐onset mild non‐atopic asthma”; Cluster 4 (n = 105), “Late‐onset asthma”; and Cluster 5 (n = 13), “Exacerbation‐prone asthma.”

**Table 2:** Univariate logistic regression analysis showing the clinical features that differed across the clusters derived by HC using the four informative features/domains (dichotomous definition of allergic sensitization)

Feature/domain	Cluster 1 (n = 132) *“Difficult asthma”*		Cluster 2 (n = 210) *“Early‐onset mild atopic asthma”*		Cluster 3 (n = 153) *“Early‐onset mild non‐atopic asthma”*		Cluster 4 (n = 105) *“Late‐onset asthma”*		Cluster 5 (n = 13) *“Exacerbation‐prone asthma”*	
	Coeff^a^ Mean (95%CI) or frequency (%)	*P*‐value	Coeff Mean (95%CI) or frequency (%)	*P*‐value	Coeff Mean (95%CI) or frequency (%)	*P*‐value	Coeff Mean (95%CI) or frequency (%)	*P*‐value	Coeff Mean (95%CI) or frequency (%)	*P*‐value
Age of Onset	**−0.03**	**.04**	**−0.10**	**<.001**	**−0.12**	**<.001**	**0.26**	**<.001**	**−**0.008	.14
Years	4.9 (2.3‐7)		4.4 (3‐6)		3.8 (2‐6)		10.7 (9‐12)		4.1 (2‐5)	
Asthma attacks	0.008	.96	**−0.04**	**.04**	**−0.04**	**.02**	**−0.04**	**.007**	**0.12**	**<.001**
Number, previous year	1.0 (0‐1)		**0.8 (0‐1)**		**0.9 (0‐1)**		**0.4 (0‐1)**		**3.5 (0‐7)**	
Allergic sensitization	**−**0.002	.88	**0.29**	**<.001**	**−0.29**	**<.001**	0.01	.26	**−**0.002	.71
Sensitized	77/132 (58%)		183/210 (87%)		27/153 (18%)		67/105 (64%)		7/13 (54%)	
Asthma severity	**0.38**	**<.001**	**−0.17**	**<.001**	**−0.13**	**<.001**	**−0.08**	**<.001**	0.006	.27
Mild	46/132 (35%)		190/210 (90%)		141/153 (92%)		91/105 (87%)		8/13 (62%)	
Moderate/severe	86/132(65%)		20/210 (10%)		12/153 (8%)		14/105 (13%)		5/13 (38%)	
Cluster stability	**1.00**		**0.99**		**0.99**		**1.00**		**1.00**	

Quantitative variables are represented as mean (95% CI). Ordinal variables are represented as frequencies (%).

a Coeff: The coefficient translates into a value of how likely a child is assigned to that cluster based on the variable response. Bolded values represent significant p‐values.

By varying the definition of allergic sensitization from the dichotomous (sensitized/not sensitized; Table 2), to ordinal (non‐atopic, monosensitized, polysensitized; Table S9) and continuous (IgE titre; Table S10), we found that the clusters remained very similar despite some changes to cluster allocation. However, the cluster stability slightly decreased.

We validated the clusters in relation to lung function (FEV_1_, FEV_1_/FVC, BDR), blood eosinophils, allergic comorbidities (eczema or rhinitis), family history, and environmental exposures (Table 3). Multinomial regression model using children in Cluster 3 (with mildest asthma) as the reference has indicated that lung function was significantly diminished only among children in Cluster 1 (“Difficult asthma”). High blood eosinophilia was a significant feature of “Difficult asthma,” “Early‐onset mild atopic asthma,” and “Late‐onset asthma” clusters, while family history of asthma and concurrent rhinitis was most common among children in “Early‐onset mild atopic asthma” cluster. Exposure to tobacco smoke was highest among children in the “Difficult asthma” cluster, although this did not reach statistical significance (*P* = .09). There was no difference in pet ownership and eczema between the clusters. Children with moderate/severe asthma were present in each of the clusters (Cluster 1, 65%; Cluster 2, 10%; Cluster 3, 8%; Cluster 4, 13%; Cluster 5, 38%).

**Table 3:** Multinomial logistic regression analysis showing lung function, blood eosinophils, tobacco smoke exposure, pet ownership, family history of asthma, and comorbidities across the five clusters derived by HC using the four informative features/domains

		Cluster 3 (n = 153) *“Early‐onset mild non‐atopic asthma”*	Cluster 1 (n = 132) *“Difficult asthma”*		Cluster 2 (n = 210) *“Early‐onset mild atopic asthma”*		Cluster 4 (n = 105) *“Late‐onset asthma”*		Cluster 5 (n = 13) *“Exacerbation‐prone asthma”*	
FEV_1%_ predicted	Mean (95%CI)	88.4 (86‐91)	83.0 (74‐91)		88.0 (80‐96)		87.9 (78‐97)		83.2 (74‐90)	
	RR (95% CI)	N/A (Reference group)	**0.68 (0.53‐0.86)**	***P*** ** < .001**	0.97 (0.79‐1.20)	*P* = .82	0.85 (0.75‐1.25)	*P* = .81	0.82 (0.39‐1.22)	*P* = .19
FEV_1_/FVC (%)	Mean (95%CI)	86.6 (85.5‐87.7)	84.8 (83.5‐86.1)		86.3 (85.4‐87.2)		85.4 (84.1‐86.8)		83.7 (78.8‐88.5)	
	RR (95% CI)	N/A (Reference group)	**0.77 (0.61‐1.09)**	***P*** ** = .03**	0.95 (0.77‐1.18)	*P* = .65	0.83 (0.65‐1.08)	*P* = .17	0.67 (0.39‐1.14)	*P* = .14
Bronchodilator reversibility (BDR), %	Mean (95%CI)	16.5 (14.7‐18.4)	18.9 (17.6‐20.2)		16.6 (14.8‐18.4)		17.5 (14.6‐20.5)		12.5 (9.4‐15.6)	
	RR (95% CI)	N/A (Reference group)	1.18 (0.93‐1.49)	*P* = .16	1.00 (0.79‐1.36)	*P* = .98	1.08 (0.83‐1.39)	*P* = .55	0.58 (0.25‐1.37)	*P* = .22
Blood eosinophils, %	Mean (95%CI)	3.2 (2.8‐3.7)	4.4 (1.8‐5.65)		5.1 (2.4‐7.1)		4.9 (2.5‐6.6)		4.2 (1.9‐4.7)	
	RR (95% CI)	N/A (Reference group)	**1.62 (1.20‐2.17)**	***P*** ** = .001**	**1.94 (1.48‐2.54)**	***P*** ** < .001**	**1.88 (1.40‐2.54)**	***P*** ** < .001**	1.51 (0.79‐2.87)	*P* = .20
Exposure to tobacco smoke	Frequency (%)	57/153 (37%)	62/132 (47%)		75/210 (36%)		41/105 (39%)		5/13 (38%)	
	RR (95% CI)	N/A (Reference group)	1.49 (0.93‐2.39)	*P* = .09	0.93 (0.61‐1.44)	*P* = .76	1.08 (0.65‐1.79)	*P* = .77	1.05 (0.32‐3.37)	*P* = .93
Pet ownership	Frequency (%)	10/153 (7%)	10/132 (8%)		15/210 (7%)		15/105 (14%)		1/13 (8%)	
	RR (95% CI)	N/A (Reference group)	1.06 (0.43‐2.58)	*P* = .90	0.99 (0.44‐2.23)	*P* = .99	2.15 (0.95‐4.89)	*P* = .07	0.006 (0.0001‐2278)	*P* = .68
Family history of asthma	Frequency (%)	35/153 (23%)	37/132 (28%)		78/210 (37%)		31/105 (30%)		3/13 (23%)	
	RR (95% CI)	N/A (Reference group)	1.13 (0.88‐1.45)	*P* = .32	**1.37 (1.11‐1.70)**	***P*** ** = .004**	1.17 (0.90‐1.52)	*P* = .23	1.01 (0.54‐1.86)	*P *= .98
Current eczema	Frequency (%)	8/153 (5%)	8/132 (6%)		17/210 (9%)		3/105 (3%)		1/13 (8%)	
	RR (95% CI)	N/A (Reference group)	1.17 (0.43‐3.21)	*P* = .76	1.59 (0.67‐3.80)	*P* = .29	0.53 (0.13‐2.06)	*P* = .36	1.51 (0.17‐13.11)	*P* = .71
Current rhinitis	Frequency (%)	42/153 (27%)	51/132 (39%)		147/210 (70%)		56/105 (53%)		6/13 (46%)	
	RR (95% CI)	N/A (Reference group)	**1.66 (1.01‐2.74)**	***P*** ** = .04**	**6.17 (3.89‐9.78)**	***P*** ** < .001**	**3.02 (1.79‐5.09)**	***P*** ** < .001**	2.26 (0.72‐7.13)	*P* = .17

Quantitative variables are represented as mean (95% CI). Ordinal variables are represented as frequencies (%); RR, relative risk; CI, confidence interval. Bolded values represent significant p‐values.

## 4 DISCUSSION

Our integrative approach of blending the data and biostatistical expertise with clinical expert domain knowledge identified a framework for the discovery of stable and clinically meaningful asthma subtypes. Using two common clustering approaches (clustering after dimensionality reduction, and using all available variables) resulted in different clusters, which were not stable. We identified four features of asthma which exemplified the differences and similarities between the clusters in our initial analyses: age of onset, allergic sensitization, asthma severity, and recent exacerbations. When we reclustered the data using these four features, the cluster stability dramatically increased, and the analysis identified five clinically meaningful asthma subtypes (early‐onset mild atopic asthma, early‐onset mild non‐atopic asthma, late‐onset asthma, difficult asthma, and exacerbation‐prone asthma).

### 4.1 Limitations/strengths

One limitation of the clustering methodologies (including our analyses) is that for the selection of variables, the investigators rely on the data which is available. The majority of previous studies used similar data sources (eg detailed questionnaire responses, sensitization, and lung function), but the variable choice for input into the model has varied.^17^ We relied on a detailed clinical assessment carried out in our study. However, we cannot exclude the possibility that some potentially important variables were not collected.

Another limitation is that our study is cross‐sectional, and precise information about the time dimension (particularly in relation to the age of onset of asthma) may be unreliable. However, cross‐sectional data sets are ideal settings for data exploration and finding latent patterns. We could test various methodologies to ascertain the most robust one for our data set. We acknowledge that adding more accurate information on onset and remission of symptoms to account for longitudinal changes could further improve asthma classification.

The strengths of our study include large number of phenotypically well‐defined patients across the spectrum of asthma severity (from mild to severe), which improves generalizability. Furthermore, to our knowledge, this is the first unsupervised analysis among children from a developing country, which offers a unique perspective on asthma subtypes in a population with different environmental exposures (and likely different genetic susceptibility) compared to studies in developed countries.

### 4.2 Interpretation

Data‐driven methods have been used in both case/patient^17^ and birth cohort studies,^15^ and are invaluable tools for discovering complex patterns and structures in data sets. However, there has been little consistency in the results between different studies and no unified methodology, leading to a degree of scepticism in the clinical community about the value of these techniques.^6^, ^18^

PCA has been used as both a stand‐alone analysis^10^, ^30^, ^31^, ^32^ and a data reduction technique prior to clustering.^8^, ^12^, ^19^, ^33^ Results from our PCA are consistent with previous studies in children, showing diversification with respect to lung function, demographics, medication use, symptom burden, and environmental factors.^7^, ^12^ One of the benefits of PCA is the reduction in dimensionality, which allows the description of the complex data using a smaller number of uncorrelated variables, while retaining as much information as possible. However, in our data set, PCA has not substantially reduced dimensionality (from a total of 47 variables, we identified 19 components with eigenvalue >1, which suggests that most variables may have been informative about different disease domains). PCA can be viewed as a method which separates signal and noise: the first dimensions extract the essential information, while the last ones are restricted to noise.^34^ Intuitively, the reduction in noise should create more stable clusters; however, in the current study, inputting the principal components into the HC model yielded unstable clusters, which suggests that PCA did not differentiate between informative and non‐informative variables. This could be a reflection of the data set or the inherent heterogeneity of the disease.

It is generally considered that there is a linear relationship between the number of variables and stability of the model. However, the clusters which emerged in the HC based on all available variables remained unstable, suggesting that it may not be useful to input all variables into the clustering algorithm, as the overloaded model may not be fully informative. Increasing the number of input variables increases the odds of the variables no longer being dissimilar (a feature important in differentiating clusters).^17^ This introduces high degrees of collinearity among the variables, making it more difficult for the model to identify unique features, and some domains may be over‐represented.

One of areas that remains to be addressed in statistical research is how to identify a meaningful set of features for cluster analysis using an unsupervised approach. In this study, we found that HC on PCA and HC on the raw data were less stable than HC on four selected features. This could be an artefact of the heterogeneity in the number of features. However, having a more meaningful semi‐automated approach to feature selection for clustering is an area of machine learning research which may have a considerable impact on understanding disease heterogeneity.

In our study, by utilizing four informative features/domains of the disease which were identified by clinical experts who interpreted the results of the unsupervised analyses markedly increased cluster stability, and the results in clinical terms appeared much more meaningful. It is likely that these domains provide important information about asthma heterogeneity, which may be lost in the noise when using all collected variables or principal components. This may be analogous to our previous findings in a population‐based birth cohort, in which dimensionality reduction suggested that of >100 item responses to validated questionnaires, only 28 were informative for the discovery of disease subtypes.^10^ Questions used to determine the presence of disease in most epidemiological studies (current wheezing, and wheezing apart from colds) were found to be redundant for understanding disease heterogeneity. This does not mean that these questions are not informative; they are key for ascertaining the presence of asthma syndrome, but are not informative when trying to uncover asthma subtypes. Thus, different domains of the disease may be required to identify disease subtypes than those used to diagnose asthma, or assess the control or response to treatment. Our results are consistent with the findings from the Childhood Asthma Management Program (which did not include children with severe asthma), which has reported that reproducible clusters with distinct clinical trajectories and different response to anti‐inflammatory medications could be differentiated based on three groups of features (atopic burden, degree of airway obstruction, and history of exacerbation).^35^

In our study, severity was one of the key features for disaggregating the asthma syndrome, but there were children with moderate/severe asthma in each of the clusters. In the US Severe Asthma Research Program (SARP), a similar HC method was used to identify four subtypes of severe asthma in childhood, differing in age of onset, lung function, FeNO, and medication use, but with an even distribution of severity among the clusters.^7^ The Trousseau Asthma Program (TAP) identified a neutrophilic‐driven severe asthma cluster that seemed to be resistant to corticosteroids.^12^ In all three studies, severe asthma was not identified as an independent cluster. Rather, severe asthmatics were present in all clusters; in TAP, the proportion of severe asthmatics ranged from 5% to 10% across the clusters,^12^ in SARP, from 61% to 84% based on ATS criteria, and from 4% to 16% according to GINA,^7^ and in our study, the occurrence of moderate/severe asthmatics ranged from 8% in Cluster 3 to 65% in Cluster 1. The results from the current and other studies suggest that severe asthma is not a single entity, but rather the extreme end of spectrum of several different asthma endotypes.

Our study identified an exacerbation‐prone cluster, which may be a separate endotype with unique underlying aetiology. A severe exacerbation cluster (which was predominantly allergy driven) was also described in the TAP cohort.^12^ Recent analysis among SARP participants (both adults and children) has suggested that exacerbation‐prone asthma may indeed be a distinct susceptibility phenotype, with implications for the targeting of exacerbation prevention strategies.^36^ Exacerbation‐prone asthma is not characterized only by asthma severity or control, and among SARP participants and in our study, a proportion of patients with exacerbation‐prone asthma had non‐severe asthma and normal lung function.^36^

The age at which a child initially wheezes has been described as a key discriminator of childhood wheeze phenotypes in multiple birth cohort studies, and our results which identified an early‐onset and a late‐onset asthma subtype are consistent with other previously published work.^20^, ^21^, ^37^ However, unlike most previous studies, we identified both an early‐onset non‐atopic subtype and an early‐onset atopic subtype.

Varying definition of allergic sensitization resulted in no material changes in our results. Using a model‐based cluster analysis, Simpson et al.^38^ have shown that sensitization comprises several different subtypes, each with unique association to asthma presence and severity, and this finding was confirmed in another birth cohort.^39^ For the prediction of future development of asthma, or asthma severity among patients with established disease, subtyping of sensitization may be crucially important.^38^, ^39^, ^41^ However, our current analysis suggests that for the purpose of asthma subtyping, a simple definition of allergic sensitization would likely suffice.

In our study, most children with asthma had normal lung function. Although lung function was significantly diminished among children in the “Difficult asthma” cluster, most patients in this cluster had normal lung function, which is consistent with other populations.^42^ Our analysis suggests that lung function may be less important for subtyping asthma, despite its perceived clinical importance for diagnosing and managing the disease. Our data also indicate that phenotyping asthma based on a single dimension of the disease (eg “eosinophilic” vs. “neutrophilic”) is unlikely to be fully informative in the search for endotypes, or for precise treatment stratification. Blood eosinophilia was a significant feature of “Difficult,” “Early‐onset mild atopic,” and “Late‐onset asthma” clusters, suggesting that there are important shared mechanisms across different asthma subtypes.^40^ Thus, while by definition each asthma endotype has a unique component in its pathophysiology,^1^, ^2^ these data indicate that some important mechanisms (eg T2‐high) overlap between most endotypes.^6^, ^40^ This may also be reflected in the responses to treatment, and patients across different endotypes may display a spectrum in responses to therapies which target shared mechanisms.^6^, ^35^

In conclusion, we identified four key features of asthma (age of onset, allergic sensitization, severity, and exacerbations in the previous year), which may be informative for ascertaining asthma subtypes. This could represent a potential future framework to facilitate the discovery of endotypes in childhood asthma. Our results highlight that factors which are key determinants of asthma presence, severity, or control may not be the most informative for determining disease endotypes.

## CONFLICT OF INTEREST

Prof. Custovic reports personal fees from Novartis, personal fees from Regeneron/Sanofi, personal fees from ALK, personal fees from Bayer, personal fees from ThermoFisher, personal fees from GlaxoSmithKline, personal fees from Boehringer Ingelheim, outside the submitted work.
