# Supplementary material for: Features of asthma which provide meaningful insights for understanding the disease heterogeneity
Source: Clin Exp Allergy. 2017 Sep 15;48(1):39–47. doi: 10.1111/cea.13014 (PMC5763358; doi:10.1111/cea.13014)
Supplement: Supplementary file 2 [file CEA-48-39-s002.docx]

**Online Supplement/ Appendix**

**Features of asthma which provide meaningful insights for understanding the disease heterogeneity**

Matea Deliu MD^1*^, Tolga S. Yavuz MD^2,3*^, Matthew Sperrin PhD^1^, Danielle Belgrave PhD^6^, Umit M. Sahiner MD^5^, Cansin Sackesen MD^4,5^, Omer Kalayci MD^5Ϯ^, Adnan Custovic MD PhD FAAAI^6Ϯ^

1. Division of Informatics, Imaging and Data Sciences, Faculty of Biology, Medicine and Health, University of Manchester, Manchester, UK

2. Gulhane School of Medicine, Department of Pediatric Allergy, Ankara, Turkey

3. Department of Paediatric Allergy, Children`s Hospital, University of Bonn, Bonn, Germany

4. Koc University, School of Medicine, Pediatric Allergy Unit, Istanbul, Turkey

5. Hacettepe University School of Medicine, Pediatric Allergy and Asthma Unit

6. Department of Paediatrics, Imperial College of Science, Technology & Medicine, London, UK

* ^Ϯ^: Equal contribution

**METHODS**

***Statistical Methods***

*Variables used*

1. Binary variables
   1. Interview-derived: sex, physician diagnosed allergic rhinitis, allergic conjunctivitis, eczema, family history of asthma, exposure to tobacco, pet ownership
   2. Medications: use of Long-acting β_2_- agonist, use of montelukast, inhaled corticosteroid dose (0, less than 400, greater than 400 *BDP equivalent to beclomethasone), use of short acting β_2_ - agonist
   3. Atopy: wheel 3mm greater than negative control to at least one of: cat, dog, cockroach, tree, weed, grass, house dust mite, moulds, serum total IgE
   4. Eosinophil % (0-0.15, 0.15-0.30, 0.3-0.5, >0.5)
2. Count variables
   1. Number of asthma exacerbations within the last 12 months, number of hospitalizations for asthma ever
3. Continuous variables
   1. Age, age of asthma onset, BMI (standardised for age, growth, sex)
   2. Lung function:
      1. FEV_1_ % predicted, FVC % predicted, FEV_1_/FVC, FEF_25-75_
      2. Bronchodilator reversibility: greater than or equal to 12% increase in FEV_1_ following administration of 200μg inhaled albuterol
      3. Airway hyperresponsiveness: concentration of methacholine required to produce a 20% decline in FEV_1_ (PC_20_) less than or equal to 8mg/ml^E1^ or greater than or equal to 10% reduction in FEV_1_ following exercise challenge^E2^.

Variables were used in their raw format apart from: (1) ‘inhaled corticosteroid use’ which was categorised with equal frequency binning and projected into a dummy variable (ICS =0, ICS <400mg, ICS>400 BDP equivalent); (2) ‘blood eosinophil count’ (<0.15, 0.15-0.30, 0.30-0.5, >0.5); (3) ‘asthma severity (mild, moderate, severe); (4) ‘asthma attacks’ (0,1,≥2); (5) ‘age of onset’(<5, 5-11, >11); and (6) ‘asthma hospitalizations’ (<2, ≥2). Our dummy variables were created using clinically meaningful categories, except for age of onset which was arbitrarily chosen. Dummy variables were only used in model 1.

Continuous variables were transformed into z-scores to simplify interpretation (whereby coefficients refer to a change of 1 standard deviation), and remove skew. No missing values were present apart from ‘methacholine challenge’ and ‘exercise challenge test’ (which were only available for approximately half of the children). These two variables were not included in the analysis.

*Model 1: Hierarchical clustering after dimensionality reduction*

Principal component analysis was performed on 45 variables in the dataset (which included aforementioned dummy variables). Variables with loadings above 0.3 on a particular principal component were chosen to represent that component. The loadings threshold was set lower than what is seen in most literature to take into account the large amount of binary data, as these naturally load lower. Orthogonal/varimax rotation was performed.

Hierarchical clustering using Ward’s method and Euclidean distance was performed on principal components. As sensitivity analysis, other distance measures (such as Minkowski, etc.) and methods (single-link, centroid, average) were also tested.

*Model 2: Hierarchical clustering using raw data*

In Model 2, dummy variables (except inhaled corticosteroid use) were removed leaving 38 raw variables. Hierarchical clustering was then performed and results were compared to the clusters identified in Model 1.

*Model 3: Identification of key stable/important features and re-clustering*

Clusters in Models 1 and 2 were generally not stable. After comparing the results, four key features were identified: age of onset, asthma severity, exacerbations, atopy. We performed a series of hierarchical cluster analyses on these four features, each time varying measures of atopy: atopic, polysensitised, monosensitized, total number of positive skin prick tests, total IgE. Specifics of atopic variables:

- - *Atopic:* binarised variable, atopic (positive skin prick test to any allergen) yes/no
  - *Sensitization:* categorical variable (0, 1, 2), 0=non atopic, 1=monosensitized (positive skin prick test to only one allergen), 2=polysensitized (positive skin prick test to more than 1 allergen)
  - *Total IgE:* quantitative variable for total IgE levels in blood (scaled)

**RESULTS**

**Figure S1.** Correlation matrix of dataset


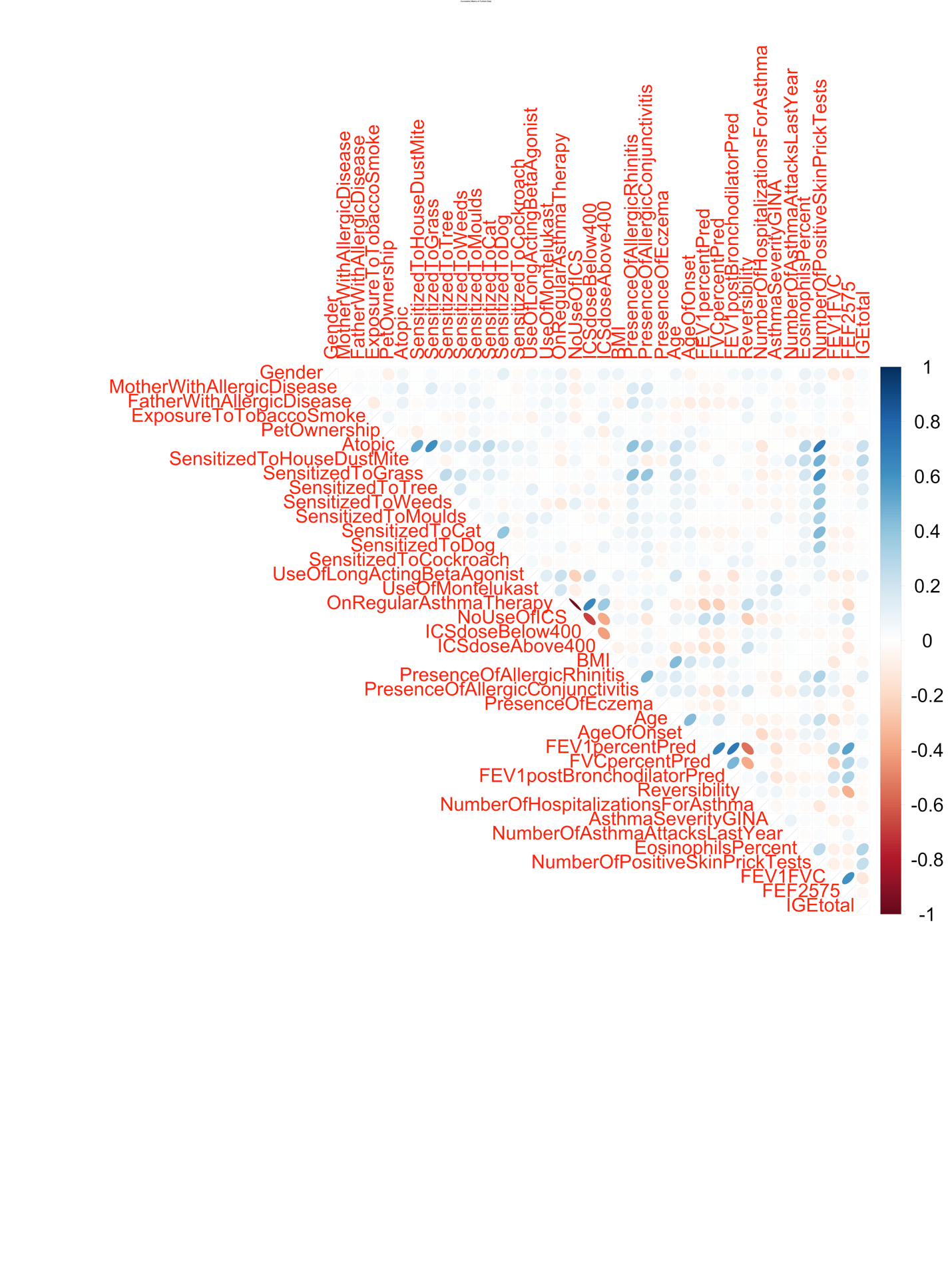


BMI = Body mass index, FEF = forced expiratory flow, ICS = inhaled corticosteroids, SPT = skin prick test. Large blue circles show high positive correlation while red circles show negative correlation. Empty boxes denote a non-significant correlation.

**Table S1.** Variables in the dataset used for analyses.

* Variables in dataset but not directly used for analysis: methacholine challenge, exercise induced fall in FEV1, number of eosinophils, total number of positive skin prick tests.

| **Variables in HC on PCA (Model 1)** | **Variables in HC on raw data (Model 2)** |
| --- | --- |
| Sex | Sex |
| Mother with allergic disease | Mother with allergic disease |
| Father with allergic disease | Father with allergic disease |
| Exposure to tobacco smoke | Exposure to tobacco smoke |
| Pet ownership | Pet ownership |
| Atopic | Atopic |
| Sensitized to house dust mite | Sensitized to house dust mite |
| Sensitized to grass | Sensitized to grass |
| Sensitized to trees | Sensitized to trees |
| Sensitized to weeds | Sensitized to weeds |
| Sensitized to moulds | Sensitized to moulds |
| Sensitized to cat | Sensitized to cat |
| Sensitized to dog | Sensitized to dog |
| Sensitized to cockroach | Sensitized to cockroach |
| Blood Eosinophil % 0.15-0.3 | Blood Eosinophil % |
| Blood Eosinophil % 0.3-0.5 | IgE total |
| Blood Eosinophil % >0.50 | Use of long-acting beta_2_ agonist |
| IgE total | Use of Montelukast |
| Use of long-acting beta_2_ agonist | Use of regular controller medication |
| Use of Montelukast | No use of inhaled corticosteroids |
| Use of regular controller medication | Inhaled corticosteroid dose <400 |
| No use of inhaled corticosteroids | Inhaled corticosteroid dose >400 |
| Inhaled corticosteroid dose <400 | BMI |
| Inhaled corticosteroid dose >400 | Presence of allergic rhinitis |
| BMI | Presence of allergic conjunctivitis |
| Presence of allergic rhinitis | Presence of eczema |
| Presence of allergic conjunctivitis | Age at follow-up |
| Presence of eczema | FEV_1_ % predicted |
| Age of onset below 5 years | FEV_1_/FVC % predicted |
| Age of onset 5-11 years | FEF 25-75 |
| Age of onset above 11 years | FEV_1_ post bronchodilator % predicted |
| FEV_1_ % predicted | Reversibility |
| FVC % predicted | FEV_1_ % predicted |
| FEV_1_/FVC % predicted | Number of hospitalizations for asthma ever |
| FEF 25-75 | Number of asthma attacks within last year |
| FEV_1_ post bronchodilator % predicted | Mild asthma |
| Reversibility | Moderate-severe asthma |
| Less than 2 hospitalizations for asthma ever | Severe asthma |
| 2 or more hospitalizations for asthma ever |  |
| No asthma attacks within last year |  |
| 1 asthma attack within last year |  |
| 2 or more asthma attacks within last year |  |
| Mild asthma |  |
| Moderate-severe asthma |  |
| Severe asthma |  |

**Table S2.** Eigenvalue and variance of the first 19 components with eigenvalues >1

|  | **Eigenvalue** | **Percentage of variance** | **Cumulative percentage of variance** |
| --- | --- | --- | --- |
| Component 1 | 4.29 | 8.95 | 8.95 |
| Component 2 | 3.19 | 6.66 | 15.61 |
| Component 3 | 2.56 | 5.34 | 20.96 |
| Component 4 | 2.34 | 4.88 | 25.84 |
| Component 5 | 2.11 | 4.40 | 30.24 |
| Component 6 | 2.09 | 4.37 | 34.62 |
| Component 7 | 1.93 | 4.04 | 38.66 |
| Component 8 | 1.86 | 3.89 | 42.55 |
| Component 9 | 1.80 | 3.76 | 46.32 |
| Component 10 | 1.69 | 3.52 | 49.84 |
| Component 11 | 1.49 | 3.11 | 52.96 |
| Component 12 | 1.39 | 2.91 | 55.87 |
| Component 13 | 1.38 | 2.87 | 58.75 |
| Component 14 | 1.21 | 2.53 | 61.29 |
| Component 15 | 1.17 | 2.45 | 63.74 |
| Component 16 | 1.15 | 2.41 | 66.16 |
| Component 17 | 1.10 | 2.30 | 68.46 |
| Component 18 | 1.07 | 2.23 | 70.69 |
| Component 19 | 1.05 | 2.19 | 72.89 |

**Table S3.** Variable loadings on first 5 components

1. Principal components 1 and 2

| **Dimension 1** | | **Dimension 2** | |
| --- | --- | --- | --- |
| **Variable** | **Loading** | **Variable** | **Loading** |
| Use of regular controller medication | 0.66 | Atopic | 0.71 |
| Moderate-severe asthma | 0.55 | Sensitized to grass | 0.65 |
| Reversibility | 0.48 | Presence of allergic rhinitis | 0.55 |
| Use of long-acting beta_2_ agonist | 0.43 | Age at follow-up | 0.51 |
| ICS dose <400 | 0.39 | Presence of allergic conjunctivitis | 0.47 |
| ICS dose > 400 | 0.33 | Less than 2 hospitalizations ever | 0.32 |
| 1 asthma attack within last year | 0.32 | Sensitized to tree | 0.32 |
| Use of Montelukast | 0.3 | Sensitized to cat | 0.31 |
| FEV_1_/FVC | -0.33 | Age of onset above 11 years | 0.3 |
| FEV_1_post bronchodilator % predicted | -0.47 | More than 2 hospitalizations for asthma ever | -0.32 |
| FVC % predicted | -0.54 | Age of onset below 5 years | -0.36 |
| FEF2575 | -0.58 |  | |
| Mild asthma | -0.62 |  |  |
| No use of ICS | -0.66 |  |  |
| FEV_1_%pred | -0.72 |  |  |

ICS: inhaled corticosteroid

1. Principal components 3 and 4

| **Dimension 3** | | **Dimension 4** |  |
| --- | --- | --- | --- |
| **Variable** | **Loading** | **Variable** | **Loading** |
| Mild asthma | 0.64 | More than 2 hospitalizations for asthma ever | 0.49 |
| Presence of allergic conjunctivitis | 0.44 | Male | 0.47 |
| Use of regular controller medication | 0.39 | FEV_1_%pred | 0.47 |
| 1 asthma attack within last year | 0.36 | FVC% pred | 0.42 |
| Reversibility | 0.35 | FEV_1_ post bronchodilator % pred | 0.4 |
| FEV_1_ post bronchodilator % pred | 0.34 | Blood Eo >0.5% | -0.3 |
| Use of long-acting beta_2_ agonist | -0.39 | Female | -0.47 |
| No use of ICS | -0.4 | Less than 2 hospitalizations for asthma ever | -0.49 |
| Moderate-severe asthma | -0.62 |  | |

1. Principal component 5

| **Dimension 5** | |
| --- | --- |
| **Variable** | **Loading** |
| No use of ICS | 0.41 |
| More than 2 hospitalizations for asthma ever | 0.33 |
| Presence of allergic rhinitis | 0.31 |
| Atopic | 0.31 |
| Blood Eo >0.50% | -0.3 |
| BMI | -0.3 |
| Less than 2 hospitalizations for asthma ever | -0.33 |
| Use of regular controller medication | -0.4 |
| ICS dose < 400 | -0.48 |

**Table S4**. Characteristics of each cluster for model 1 (HC after PCA dimensionality reduction).

Quantitative variables are represented as mean (standard deviation and interquartile range). Binary and categorical variables are represented as proportions (%). Binary and categorical variables are calculated using chi-squared test of significance; continuous variables are calculated using Kruskal-Wallis test of significance.

| **Variable** | | **Cluster 1**  **n = 102** | **Cluster 2**  **n = 70** | **Cluster 3**  **n = 117** | **Cluster 4**  **n = 149** | **Cluster 5**  **n = 175** | **p-value** |
| --- | --- | --- | --- | --- | --- | --- | --- |
| Sex | Male | 61/102, 59% | 56/70, 80% | 84/117, 72% | 72/149, 48% | 95/175, 54% | <0.001 |
|  | Female | 41/102, 41% | 14/70, 20% | 33/117, 28% | 77/149, 52% | 81/175, 46% | <0.001 |
| Mother with allergic disease | | 8/102, 7% | 19/70, 28% | 36/117, 31% | 30/149, 20% | 24/175, 15% | 0.002 |
| Father with allergic disease | | 6/102, 5% | 40/70, 57% | 35/117, 30% | 20/149, 13% | 19/175, 11% | 0.01 |
| Exposure to tobacco smoke | | 28/102, 27% | 52/70, 74% | 52/117, 44% | 60/149, 40% | 48/175, 27% | 0.15 |
| Pet ownership | | 7/102, 6% | 37/70, 53% | 16/117, 14% | 23/149, 15% | 12/175, 7% | 0.28 |
| Atopic | | 75/102, 75% | 31/70, 48% | 105/117, 89% | 49/149, 32% | 102/175, 58% | <0.001 |
| Sensitized to house dust mite | | 45/102, 41% | 7/70, 10% | 25/117, 21% | 22/149, 15% | 65/175, 37% | <0.001 |
| Sensitized to grass | | 30/102, 9% | 17/70, 25% | 90/117, 77% | 19/149, 13% | 56/175, 32% | <0.001 |
| Sensitized to trees | | 7/102, 6% | 0/70 | 15/117, 13% | 2/149, 1% | 5/175, 3% | <0.001 |
| Sensitized to weeds | | 5/102, 5% | 0/70 | 7/117, 6% | 0/149 | 14/175, 8% | 0.008 |
| Sensitized to moulds | | 8/102, 7% | 2/70, 3% | 12/117, 10% | 4/149, 3% | 12/175, 7% | 0.03 |
| Sensitized to cat | | 11/102, 10% | 0/70 | 21/117, 18% | 2/149, 1% | 15/175, 9% | <0.001 |
| Sensitized to dog | | 3/102, 3% | 1/70, 1% | 9/117, 8% | 0/149 | 2/175, 1% | <0.001 |
| Sensitized to cockroach | | 2/102, 2% | 0/70 | 3/117, 3% | 1/149, 1% | 5/175, 3% | 0.6 |
| Blood Eosinophil % 0.15-0.3 | | 8/102, 8% | 9/70, 13% | 0/117 | 1/149, 1% | 0/175 | 0.5 |
| Blood Eosinophil % 0.3-0.5 | | 17/102, 16% | 16/70, 23% | 8/117, 7% | 10/149, 7% | 4/175, 2% | 0.006 |
| Blood Eosinophil % >0.50 | | 83/102, 81% | 44/70, 64% | 109/117, 93% | 130/149, 87% | 173/175, 99% | <0.001 |
| IgE total | | 365.5 (534.9, 34-440.3) | 79.1 (115.5, 11.5-83.5) | 293.8 (582, 49-272) | 108.8 (276.1,12-106) | 233.1 (476,42-221.5) | <0.001 |
| Use of long-acting beta_2_ agonist | | 43/102, 41% | 4/70, 6% | 3/117, 3% | 0/149 | 0/175 | <0.001 |
| Use of Montelukast | | 24/102, 23% | 10/70, 14% | 8/117, 7% | 2/149, 0.01% | 7/175, 4% | <0.001 |
| Use of regular controller medication | | 76/102, 72% | 61/70, 88% | 105/117, 89% | 133/149, 90% | 10/175, 6% | <0.001 |
| No use of inhaled corticosteroids | | 30/102, 29% | 8/70, 12% | 10/117, 9% | 15/149, 10% | 174/175, 99% | <0.001 |
| Inhaled corticosteroid dose <400 | | 55/102, 52% | 44/70, 64% | 74/117, 63% | 83/149, 56% | 6/175, 3% | <0.001 |
| Inhaled corticosteroid dose >400 | | 22/102, 22% | 10/70, 14% | 33/117, 28% | 49/149, 33% | 1/175, 0.01% | <0.001 |
| BMI | | 18.6 (3.6, 16-22.2) | 19.2 (3.9, 16.3-22.2) | 16.9 (3.1, 15.8-19.1) | 17.5 (3.4, 15.4-19.2) | 17.9 (3.4, 15.9-21.1) | 0.08 |
| Presence of allergic rhinitis | | 50/102, 50% | 15/70, 21% | 92/117, 78% | 35/149, 24% | 110/175, 63% | <0.001 |
| Presence of allergic conjunctivitis | | 6/102, 6% | 6/70, 9% | 71/117, 61% | 8/149, 5% | 21/175, 12% | <0.001 |
| Presence of eczema | | 9/102, 9% | 3/70, 4% | 11/117, 9% | 6/149, 4% | 8/175, 5% | 0.35 |
| Age of onset below 5 years | | 39/102, 39% | 19/70, 19% | 25/117, 21% | 88/149, 61% | 64/175, 36% | <0.001 |
| Age of onset 5-11 years | | 38/102, 37% | 56/70, 81% | 30/117, 26% | 56/149, 38% | 95/175, 54% | 0.02 |
| Age of onset above 11 years | | 25/102, 24% | 1/70, 1% | 62/117, 53% | 3/149, 2% | 16/175, 9% | 0.02 |
| FEV_1_ % predicted | | 75.6 (10.9, 70.25-82) | 93.8 (11.7, 86-102) | 82 (11.8, 74-90) | 83.8 (11.2, 77-90) | 96.7 (11.3, 90-104) | <0.001 |
| FVC % predicted | | 89.9 (14.4,81-99.8) | 100.4 (12.7, 91-111.8) | 90.4 (16.9, 81-102) | 92 (11.4, 84-99) | 105.1 (11.3, 97-112) | <0.001 |
| FEV_1_/FVC % predicted | | 80.8 (8.8, 74.3-86.8) | 86.7 (6.0, 78.7-90.3) | 86.2 (5.6, 78.6-90.2) | 86.8 (5.9, 85.7-91.2) | 87.6 (6.8, 85-92) | <0.001 |
| FEF 25-75 | | 88.3 (11.2, 83-96) | 82 (21.9, 76-88) | 72.4 (17.7, 61-84) | 84.6 (22.9, 77-89.1) | 97.5 (6.8, 80-111) | <0.001 |
| FEV_1_ post bronchodilator % predicted | | 65.3(22.8, 50-75.5) | 108.1 (12.4, 101-115) | 100.2 (10.7, 99.7-105.2) | 100.3 (11.9, 99.8-108.2) | 105.2 (12.6, 97-113.3) | <0.001 |
| Reversibility | | 17.9 (10.1, 8.1-18.6) | 18.7 (10.3, 9.1-19.6) | 24.4 (18.1, 13.1-29.3) | 18.1 (13.7, 8.2-21.8) | 9.8 (12.6, 0-14.4) | <0.001 |
| Less than 2 hospitalizations for asthma ever | | 104/102, 99% | 40/70, 58% | 0/117 | 0/149 | 171/175, 98% | <0.001 |
| 2 or more hospitalizations for asthma ever | | 1/102, 1% | 29/70, 42% | 0/117 | 0/149 | 1/175, 0.6% | <0.001 |
| No asthma attacks within last year | | 54/102, 53% | 29/70, 42% | 71/117, 61% | 96/149, 64% | 140/175, 80% | <0.001 |
| 1 asthma attack within last year | | 15/102, 15% | 14/70, 20% | 42/117, 36% | 37/149, 25% | 13/175, 7% | <0.001 |
| 2 or more asthma attacks within last year | | 23/102, 22% | 27/70, 39% | 4/117, 3% | 16/149, 11% | 22/175, 13% | <0.001 |
| Mild asthma | | 2/102, 2% | 59/70, 85% | 103/117, 88% | 142/149, 97% | 174/175, 99% | <0.001 |
| Moderate-severe asthma | | 95/102, 90% | 10/70, 14% | 14/117, 11% | 6/149, 3% | 1/175, 1% | <0.001 |
| Severe asthma | | 8/102, 8% | 1/70, 1% | 1/117, 1% | 1/149, 1% | 0/175 | <0.001 |

**Table S5**. Characteristics of each cluster for model 2 (HC using all available variables).

Quantitative variables are represented as mean (standard deviation and interquartile range). Binary and categorical variables are represented as proportions (%). Binary and categorical variables are calculated using chi-squared test of significance; continuous variables are calculated using Kruskal-Wallis test of significance.

| **Variable** | | **Cluster 1**  **n = 168** | **Cluster 2**  **n = 100** | **Cluster 3**  **n = 103** | **Cluster 4**  **n = 223** | **Cluster 5**  **n = 19** | **p-value** |
| --- | --- | --- | --- | --- | --- | --- | --- |
| Sex | Male | 76/168, 55% | 62/100, 62% | 72/103, 70% | 149/223, 67% | 13/19, 68% | 0.07 |
|  | Female | 92/168, 45% | 38/100, 38% | 31/103, 30% | 74/223, 33% | 6/19, 32% | 0.07 |
| Mother with allergic disease | | 43/168, 26% | 24/100, 24% | 16/103, 16% | 32/223, 14% | 4/19, 21% | 0.04 |
| Father with allergic disease | | 28/168, 17% | 12/100, 12% | 17/103, 17% | 24/223, 11% | 2/19, 11% | 0.40 |
| Exposure to tobacco smoke | | 78/168, 46% | 36/100, 36% | 47/103, 46% | 72/223, 32% | 7/19, 37% | 0.03 |
| Pet ownership | | 10/168, 6% | 13/100, 13% | 9/103, 9% | 18/223, 8% | 1/19, 5% | 0.41 |
| Atopic | | 110/168, 65% | 81/100, 81% | 68/103, 67% | 86/223, 39% | 16/19, 84% | <0.001 |
| Sensitized to house dust mite | | 52/168, 31% | 39/100, 39% | 31/103, 31% | 42/223, 19% | 13/19, 68% | <0.001 |
| Sensitized to grass | | 64/168, 38% | 58/100, 58% | 44/103, 43% | 52/223, 23% | 9/19, 47% | <0.001 |
| Sensitized to trees | | 9/168, 5% | 7/100, 7% | 5/103, 5% | 5/223, 2% | 2/19, 11% | 0.81 |
| Sensitized to weeds | | 8/168, 5% | 3/100, 3% | 3/103, 3% | 12/223, 5% | 1/19, 5% | 0.20 |
| Sensitized to moulds | | 14/168, 8% | 6/100, 6% | 5/103, 5% | 12/223, 5% | 1/19, 5% | 0.75 |
| Sensitized to cat | | 13/168, 8% | 8/100, 8% | 16/103, 16% | 13/223, 5% | 1/19, 5% | 0.06 |
| Sensitized to dog | | 5/168, 3% | 1/100, 1% | 6/103, 6% | 4/223, 2% | 0/19 | 0.17 |
| Sensitized to cockroach | | 3/168, 2% | 2/100, 2% | 3/103, 3% | 3/223, 1% | 0/19 | 0.85 |
| Blood Eosinophil % | | 2.0 (3.4, 0.7-5.8) | 4.1 (2.4, 1.7-6.7) | 2.8 (3.1, 1.1-6.1) | 2.2 (2.1, 0.8-4.5) | 6.1 (4.5, 2.1-15.7) | 0.23 |
| IgE total | | 136.1 (167.4, 24.8-172.5) | 240.6 (296.2, 55.7-245) | 250.5 (294.9, 31-345.5) | 121.4 (153.4, 21-157.5) | 2152.9 (1005.3, 1680-2169) | <0.001 |
| Use of long-acting beta_2_ agonist | | 19/168, 11% | 8/100, 8% | 12/103, 12% | 7/223, 3% | 5/19, 26% | <0.001 |
| Use of Montelukast | | 20/168, 12% | 7/100, 7% | 9/103, 9% | 14/223, 6% | 1/19, 5% | 0.33 |
| Use of regular short acting beta_2_ agonist | | 134/168, 80% | 62/100, 62% | 76/103, 76% | 98/223, 44% | 14/19, 74% | <0.001 |
| No use of inhaled corticosteroids | | 34/168, 20% | 41/100, 41% | 27/103, 27% | 130/223, 58% | 5/19, 26% | <0.001 |
| Inhaled corticosteroid dose <400 | | 82/168, 49% | 52/100, 52% | 52/103, 50% | 66/223, 30% | 10/19, 53% | <0.001 |
| Inhaled corticosteroid dose >400 | | 51/168, 30% | 7/100, 7% | 24/103, 23% | 27/223, 12% | 4/19, 21% | <0.001 |
| BMI | | 18.2 (3.8, 15.5-20.1) | 18.3 (3.6, 15.5-20.9) | 17.9 (3.5, 15.7-19.9) | 18.8 (3.4, 16.1-21.3) | 19.2 (3.6, 15.7-22.4) | <0.001 |
| Presence of allergic rhinitis | | 88/168, 52% | 69/100, 69% | 50/103, 49% | 84/223, 38% | 11/19, 58% | <0.001 |
| Presence of allergic conjunctivitis | | 39/168, 23% | 34/100, 34% | 20/103, 20% | 17/223, 8% | 2/19, 11% | <0.001 |
| Presence of eczema | | 12/168, 7% | 3/100, 3% | 8/103, 8% | 13/223, 6% | 1/19, 5% | 0.63 |
| Age at follow-up | | 3.1 (2.1, 2.5-4.8) | 11.2 (3, 7-10.7) | 5.8 (2.1, 1.8-7.2) | 9.2 (2.7, 7.2-10.7) | 6.6 (2.2, 3.4-8.2) | <0.001 |
| FEV_1_ % predicted | | 79.92 (10.8, 74-87) | 94.0 (10.7, 85.8-101.3) | 72.6 (9.8, 68-78) | 95.2 (9.5, 87-102) | 91.2 (17.3, 80.5-99.5) | <0.001 |
| FVC % predicted | | 85.6 (11.5, 78-93.25) | 106.0 (14.6, 94.8-115.3) | 89.1 (15.9, 81-100) | 102.1 (9.5, 94.5-108) | 102.2 (15.1, 94-109.5) | <0.001 |
| FEV_1_/FVC % predicted | | 88.9 (5.3, 85.8-92) | 85.7 (6.1, 83-90) | 78.1 (7.3, 73-83) | 87.3 (5.3, 84-90) | 84.6 (6.9, 81-89) | <0.001 |
| FEF 25-75 | | 81.8 (22.8, 68-90) | 86.2 (24.2, 70-103) | 54.8 (13.6, 45.5-61) | 92.8 (21.4, 76.5-107) | 82.5 (22.5, 69-89) | <0.001 |
| FEV_1_ post bronchodilator % predicted | | 92.8 (11.4, 86-100.8) | 106.2 (10.8, 98.5-112) | 94.2 (12.1, 86-101) | 105.7 (11.5, 97-113) | 103.6 (18.9, 92.3-105.8) | <0.001 |
| Reversibility | | 16.6 (6.3, 12.9-20) | 14.3 (7.1, 12.1-17.9) | 31.6 (21.8, 14.1-43.2) | 11.9 (6.8, 5.15-15.7) | 14.6 (6.3, 12.5-15.8) | <0.001 |
| Number of hospitalizations for asthma ever | | 0.8 (0.2, 0.4-2.2) | 0.4 (0.2, 0-1.1) | 0.5 (0.3, 0.1-1.9) | 0.1 (0.2, 0-1.8) | 0.1 (0.2, 0-1) | 0.63 |
| Number of asthma attacks within last year | | 0.8 (1.8, 0.4-1.7) | 0.6 (2.3, 0-7) | 0.7 (2.3, 0.2-1.3) | 0.2 (1.7, 0.1-1.3) | 2.5 (2.2, 0.9-4.8) | <0.001 |
| Mild asthma | | 117/168, 69% | 92/100, 92% | 47/103, 46% | 202/223, 90% | 11/19, 58% | <0.001 |
| Moderate-severe asthma | | 43/168, 26% | 8/100, 8% | 54/103, 52% | 20/223, 9% | 8/19, 42% | <0.001 |
| Severe Asthma | | 8/168, 5% | 0/100 | 2/103, 2% | 1/223, 1% | 0/19 | 0.01 |

**Table S6**. Crosstabs of cluster subject allocation. Results are presented as proportions.

The columns represent the cluster membership from the analysis done on principal components. Rows

represent cluster membership from the analysis done on raw data. Highlighted values indicate highest overlap

and likely corresponding clusters. Association was measured by the chi-squared test.

|  | **HC after PCA dimensionality reduction** | | | | | |  |
| --- | --- | --- | --- | --- | --- | --- | --- |
| **HC using all available variables** |  | Cluster 1 | Cluster 2 | Cluster 3 | Cluster 4 | Cluster 5 | Total |
|  | Cluster 1 | 37 (22%) p=0.26 | 17 (10%) P<0.001 | 34 (20%) p=0.02 | **65 (39%) p<0.001** | 15 (9%) p<0.001 | 168 |
|  | Cluster 2 | 5 (5%), p=0.82 | 6 (6%) p=0.06 | **45 (45%) p<0.001** | 8 (8%) p<0.001 | 36 (36%)  p<0.001 | 100 |
|  | Cluster 3 | **45 (44%) p<0.001** | 8 (8%) p=0.01 | 29 (28%) p=0.22 | 16 (15%) p=0.008 | 5 (5%) p<0.001 | 103 |
|  | Cluster 4 | 8 (4%), p0<0.001 | 37 (16%) p=0.002 | 6 (3%) p<0.001 | 58 (26%) p=0.51 | **114 (51%) p<0.001** | 223 |
|  | Cluster 5 | 7 (37%)  p<0.001 | **2 (11%) p<0.001** | 3 (16%) p=0.001 | 2 (11%) p=0.04 | 5 (26%) p<0.001 | 19 |
| Total |  | 102  17% | 70  11% | 117  19% | 149  24% | 175  29% | **613**  **100%** |

**Table S7.** Clinical comparison of outcomes based on the univariate analysis

The bolded parts indicate the major differences between the two results

| **HC after PCA dimensionality reduction** | **HC using all available variables** |
| --- | --- |
| **Cluster 1**   - *Moderate-severe asthma* - *Diminished lung function* - ***2 or more exacerbations, less than 2 hospitalizations*** - ***High IgE*** - High medication use   - Mild eosinophilia | **Cluster 3**   - *Moderate-severe asthma* - *Diminished lung function* - ***1 attack*** - ***Sensitized to cat, dog and tree*** - Reversible airways |
| **Cluster 2**   - *Frequent hospitalizations* - *Frequent attacks* - *Late-onset* - Moderate eosinophilia - Moderate medication use - Male - Family history - Normal lung function | **Cluster 5**   - *Frequent attacks* - *Late-onset* - Atopic - High IgE - On LABA |
| **Cluster 3**   - *Late- onset* - *Multiple atopy* - *High BMI* - *Allergic rhinitis/conjunctivitis* - *Mild asthma* - 1 attack, less than 2 hospitalizations - ***Diminished lung function and high reversibility*** - Male - Normal eosinophils - Family history - Exposure to tobacco - Low medication use | **Cluster 2**   - *Late- onset* - *Multiple atopy* - *High BMI* - *Allergic rhinitis/conjunctivitis* - *Mild asthma* - No attacks - ***Good lung function*** |
| **Cluster 4**   - *Early onset* - *Female* - *Non atopic* - *High steroid doses* - *Slightly diminished lung function* - ***Mild asthma*** - 1 attack per year | **Cluster 1**   - *Early onset* - *Female* - *Slightly atopic; lower total IgE* - *High steroid and medication use* - *Diminished lung function* - ***Severe asthma*** - Family history - Exposed to tobacco |
| **Cluster 5**   - *Mild asthma* - *Good lung function* - *Low medication use* - ***Sensitized to HDM, weed*** - *No attacks* - *Less than 2 hospitalizations* - Hypereosinophilic | **Cluster 4**   - *Mild asthma* - *Good lung function* - *Low medication use* - ***Non-atopic*** - ***Low IgE*** - Male |

**Table S8.** Cluster stability for the HC after PCA dimensionality reduction, HC using all available variables, and HC using the “informative” subset of features.

The mean values for the bootstrapping samples indicating stability. Good stability is considered to have a bootstrap mean>0.75. Stable clusters highlighted in bold: HC on principal components producing only one stable cluster (Cluster 1), HC using all available data producing two stable clusters (Clusters 2 and 5), while in HC using the “informative” subset of features, all clusters were stable.

| **HC after PCA dimensionality reduction** | **HC using all available variables** | **HC using the “informative” subset of features** |
| --- | --- | --- |
| **Cluster 1: 0.98**  Cluster 2: 0.23  Cluster 3: 0.41  Cluster 4: 0.60  Cluster 5: 0.19 |  |  |
|  | Cluster 1: 0.59  **Cluster 2: 0.82**  Cluster 3: 0.53  Cluster 4: 0.61  **Cluster 5: 0.86** |  |
|  |  | **Cluster 1: 1.00**  **Cluster 2: 0.99**  **Cluster 3: 0.99**  **Cluster 4: 1.00**  **Cluster 5: 1.00** |

**Table S9.** Univariate logistic regression analysis using sensitization status as ordinal variable (non-atopic, monosensitized, polysensitized)

*Coeff: The coefficient translates into a value of how likely a child is assigned to that cluster based on the variable response.

| **Variable** | **Cluster 1 (n=132)** | | **Cluster 2 (n=135)** | | **Cluster 3 (n=181)** | | **Cluster 4 (n=151)** | | **Cluster 5 (n=14)** | | |
| --- | --- | --- | --- | --- | --- | --- | --- | --- | --- | --- | --- |
|  | Coeff | p-value | Coeff | p-value | Coeff | p-value | Coeff | p-value | Coeff | p-value |  |
| **Age of Onset** | **-0.03** | **0.04** | **-0.15** | **<0.001** | **0.31** | **<0.001** | **-0.12** | **<0.001** | -0.006 | 0.25 |  |
| **Asthma attacks** | 0.0008 | 0.96 | **-0.03** | **0.04** | **-0.05** | **0.009** | **-0.04** | **0.01** | **0.12** | **<0.001** |  |
| **Sensitization status** | -0.004 | 0.79 | **0.15** | **0.003** | **0.10** | **<0.001** | **-0.26** | **<0.001** | 0.004 | 0.45 |  |
| **Asthma Severity** | **0.39** | **<0.001** | **-0.11** | **<0.001** | **-0.15** | **<0.001** | **-0.12** | **<0.001** | 0.005 | 0.35 |  |
| **Cluster Stability** | **0.98** | | **0.64** | | **0.57** | | **0.82** | | **0.83** | |  |

**Table S10.** Univariate logistic regression analysis using sensitization as continuous variable (IgE titer)

*Coeff: The coefficient translates into a value of how likely a child is assigned to that cluster based on the variable response.

| **Variable** | **Cluster 1 (n=132)** | | **Cluster 2 (n=339)** | | **Cluster 3 (n=109)** | | **Cluster 4 (n=18)** | | **Cluster 5 (n=14)** | |
| --- | --- | --- | --- | --- | --- | --- | --- | --- | --- | --- |
|  | Coeff | p-value | Coeff | p-value | Coeff | p-value | Coeff | p-value | Coeff | p-value |
| **Age of Onset** | **-0.03** | **0.03** | **-0.21** | **<0.001** | **0.25** | **<0.001** | -0.00005 | 0.99 | -0.004 | 0.48 |
| **Asthma attacks** | 0.005 | 0.74 | **-0.10** | **<0.001** | **-0.03** | **0.02** | -0.003 | 0.52 | **0.13** | **<0.001** |
| **Total IgE** | -0.003 | 0.86 | **-0.06** | **0.003** | -0.05 | 0.01 | **0.11** | **<0.001** | 0.0005 | 0.99 |
| **Asthma Severity** | **0.39** | **<0.001** | **-0.29** | **<0.001** | **-0.09** | **<0.001** | -0.01 | 0.05 | -0.01 | 0.86 |
| **Cluster Stability** | **0.97** | | **0.81** | | **0.67** | | **0.70** | | **0.67** | |

**References**

S1. Sekerel BE, Saraclar Y, Kalayci O, Cetinkaya F, Tuncer A, Adalioglu G. Comparison of four different measures of bronchial responsiveness in asthmatic children. Allergy 1997;52:1106-9.

S2. Joos GF, O'Connor B, Anderson SD, et al. Indirect airway challenges. Eur Respir J 2003;21:1050-68.

S3. C H. Cluster-wise assessment of cluster stability. London UK: University College London; 2006.
